# P-1029. Ambulatory CLABSIs in a Pediatric Hematology Oncology Population

**DOI:** 10.1093/ofid/ofaf695.1225

**Published:** 2026-01-11

**Authors:** Cara E Charnogursky, Rebecca A Stern, Thomas R Talbot, Ritu Banerjee

**Affiliations:** Vanderbilt University Medical Center, Nashville, TN; Vanderbilt University Medical Center, Nashville, TN; Vanderbilt University Medical Center, Nashville, TN; Vanderbilt University Medical Center, Nashville, TN

## Abstract

**Background:**

Central line-associated blood stream infections (CLABSIs) lead to prolonged hospital stays, increased healthcare costs, and contribute significantly to patient morbidity and mortality. Inpatient CLABSIs are routinely tracked by hospitals; however, CLABSIs that occur in the ambulatory setting are understudied.

**Figure:**
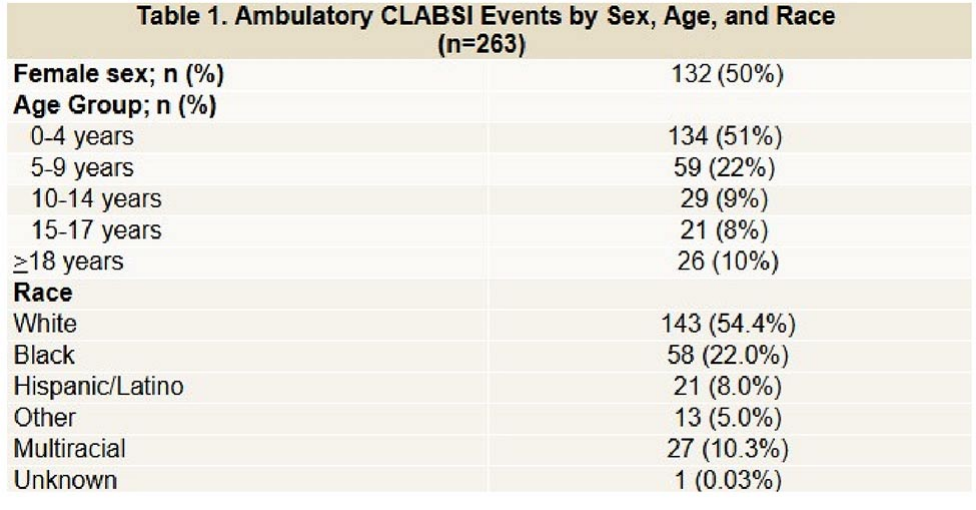

**Figure:**
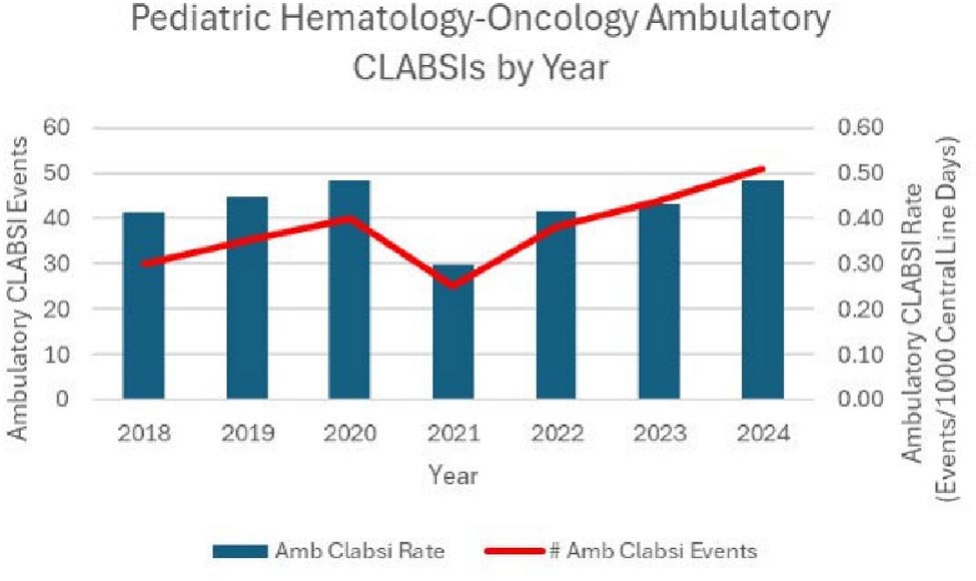

**Methods:**

We developed an electronic dashboard to capture ambulatory CLABSIs in a cohort of pediatric hematology oncology patients seen at an academic medical center from January 1, 2018 through December 31, 2024. Patients evaluated in select ambulatory clinics during the calendar year were included if they had a central line in place and had a positive blood culture that occurred in the ambulatory setting, Emergency Department, or on day 1 or 2 of hospital admission. Central line days were calculated based on insertion and removal dates as documented in the electronic medical record. We describe ambulatory CLABSIs by sex, age, race, and causative organism.

**Results:**

There were 263 total ambulatory CLABSIs observed over 616,176 central line days with a rate of 0.427 CLABSIs per 1,000 central line days. Ambulatory CLABSI events by sex, age, and race are described in Table 1. The number of ambulatory CLABSIs were highest in the 0-4 year age group and in white children. Ambulatory CLABSI events and rates over time are shown in Figure 1. The ambulatory CLABSI rate was lowest in 2021 and increased in subsequent years. The most common causative organism was Staphylococcus epidermidis (36/263, 13.7%), followed by Escherichia coli (23/263, 8.7%), Klebsiella pneumoniae (21/263, 8.0%), Staphylococcus aureus (18/263, 6.8%), and Enterobacter cloacae complex (16, 6.1%).

**Conclusion:**

Ambulatory CLABSIs may cause significant morbidity and mortality in the pediatric population and better characterization of these events may help guide targeted interventions and surveillance strategies. Future analysis will assess risk factors for the development of ambulatory CLABSI.

**Disclosures:**

Thomas R. Talbot, III, MD, MPH, OmniSolve: Board Member

